# Supplementary material for: IPS (In-Plant System) Delivery of Double-Stranded Vitellogenin and Vitellogenin receptor via Hydroponics for Pest Control in Diaphorina citri Kuwayama (Hemiptera: Psyllidae)
Source: Int J Mol Sci. 2023 May 30;24(11):9497. doi: 10.3390/ijms24119497 (PMC10253278; doi:10.3390/ijms24119497)
Supplement: Supplementary file 1 [file ijms-24-09497-s001.zip › Supplementary Materials.pdf]

Supplementary Materials

1 Methods of dsRNA synthesis

The purified PCR *Vg4* and *VgR* fragments were cloned into the pMD18T vector (DH5α, Thermo Fisher Scientific, USA). The sequencing results showed that interfering fragments of *Vg4* and *VgR* genes were successfully cloned into pMD18T vector.

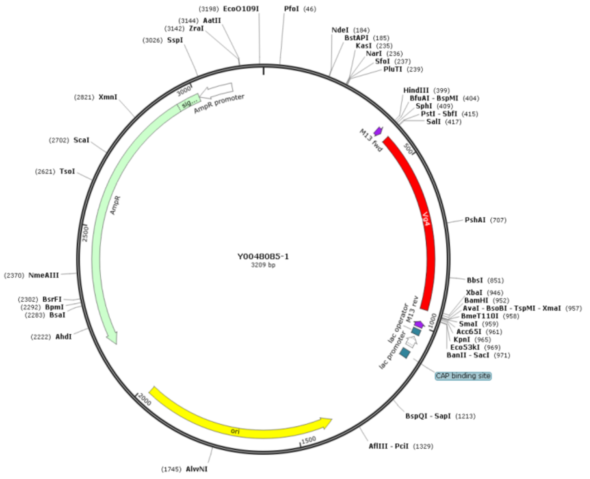

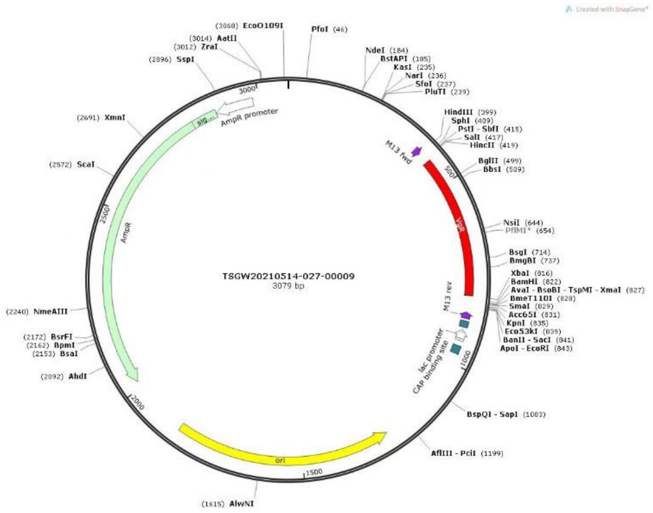

The resulted *Vg4* plasmids were used as templates to generate *dsVg4* (477bp) with the dsRNA synthesis kit (RNAsyn Biottech Co., Ltd, China). The principle of dsRNA synthesis kit was added GGG and CCC gene fragments as the promoters of dsRNA synthesis respectively before the first base of target gene segment and the end base after, separately synthesize the positive strand RNA and negative strand RNA of the target gene, design primers based on this gene fragment, and then mix the positive strand RNA and negative strand RNA in equal proportions to conduct annealing reaction to obtain dsRNA. Synthesized dsRNA was quantitated by a N80 Touch nanophotometer (Implen, Germany) at 260 nm, and the integrity was analysed by agarose gel

electrophoresis. The syntheses of *dsVgR* (346 bp) were carried out as described for *dsVg4* (Table 1). It should be noted that *dsGPF* (726 bp) was directly synthesized with the *GFP* of pUC18 plasmid as the template, plasmid was prepared in advance by the laboratory, so the designed primer adds T7 promoter fragment, and the dsRNA synthesis kit was still used.

The dsRNA synthesis method is as follows:

1.1 Transcription template obtained by PCR amplification

(1) PCR amplification reaction system

F	1μl
R	1μl
Plasmid template	1μl (20-50ng)
2×Tolo FastPfu Premix	25μl
Add deionized water to	50μl

(2) PCR amplification reaction conditions

temperature	time	circulate
**94℃**	5min	**1**
**94℃**	30s	
**52-62℃**	2Kb/15s	**30**
**72℃**	…	
**72℃**	10min	**1**
**4℃**	∞	

For electrophoresis detection and recovery of amplified fragments, please refer to the instructions of the kit (*EasyPure*® Quick Gel Extraction Kit, TransGen Biotech, China).

1.2 DsRNA synthesis

The RNA synthesis system is as follows：

**Name of ingredients**	**Dosage**	**Final concentration**
DEPC water	51.6 μL	
5xTranscription buffer	24 μL	
NTP	20.4 μL	Each 0.5mM
Transcriptional template	6 μL	5ug
RNase enzyme inhibitor	3.6 μL	1U/μl
DTT	2.4 μL	5mM
RNA polymerase	12 μL	
Total volume	120 μL	

Transcription at 37 ℃ for 1 h, then add 12 μL DNaseI, react at 37 ℃ for 30 min, remove the template, and then purify it with the RNA purification kit of NEB to obtain positive strand RNA and negative strand RNA; the positive strand RNA and negative strand RNA were mixed in equal proportion and annealed to obtain dsRNA.

1.3 Agarose gel electrophoresis detection

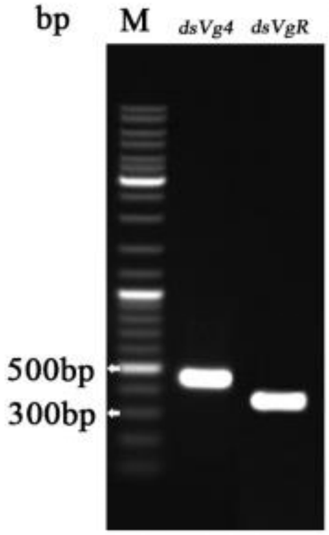

Result analysis: The dsRNA product band was clear, consistent with the expected size, and has no obvious degradation.

1.4 RT Reaction

cDNA synthesis of *dsVg4* and *dsVgR* was carried out using the PrimescriptTM RT reagent kit with gDNA eraser (perfect real time) (TaKaRa, Japan).

PCR amplification reaction

PCR amplification reaction system

F	1μl
R	1μl
cDNA template	1μl
2×Tolo FastPfu Premix pfu	25μl
Add deionized water to	50μl

PCR amplification reaction conditions

Temperature	Time	loop
**94℃**	5min	**1**
**94℃**	30s	
**55℃**	2Kb/15s	**30**
**72℃**	…	
**72℃**	10min	**1**
**4℃**	∞	

Agarose gel electrophoresis detection

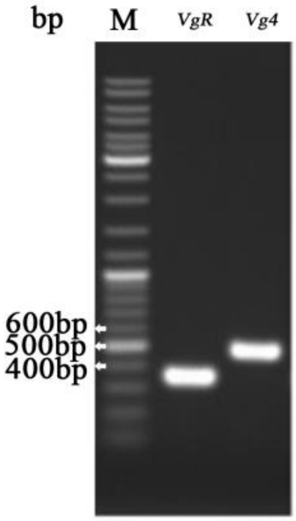

Result analysis: *DsVg4* and *dsVgR* were used as templates to clone *Vg4* and *VgR* gene products with clear bands, consistent with the expected size.

2 Comparison of sequencing results

Vg4 sequencing base sequence, the sequencing result was basically consistent with the base sequence of *Vg4* gene.

AATGGCAGTCGAGGAGAGAGCTGCTGATTATTGGCTGTTCTCCCCAACACCGCCAGATACCCAACCCG
TGAATACATCAAGGAATTCTTCAACCTTGCCACCAGCTCTCAAGTCACCAAACAAGCTCACCTCAACA
CCTCTGCTATCCTCTCCGTCGCCAGTTTGGCAAGAAAAGCCCAAGTTGACTCTGACAACTCACACAAC
CAATACCCAGTTCATGCTTTCGGACCCCTGTCATCCAAAAACAGCAAAGACATCACTGAAAGATACAT
CCCATACTTGGCCAACAAACTCAAGGAAGCTACCAGAAACCAAGACAGTCTGAAAGCTCAAGTCTAC
ATCAAAGCTCTCGGAAACTTAGGACATACCGCCGTCCTCGCTGTCTTCAAACCATACCTTGAAGGAAA
AGCCCCAGCCACCAACTTCCAACGTCTTTCATGGGTTGCCCAAAACA

The original template of *Vg4* gene for synthesizing *dsVg4* (*Vitellogenin-A1-like*, *Diaphorina citri Vg4*, 477bp).

ATGGCCATGAAACAATGGATTGAATCCGGCAAAGTCGAAGGAGAAGAAGCTGCTGAATTATTGGCTGT
TCTCCCCAACACCGCCAGATACCCAACCCGTGAATACATCAAGGAATTCTTCAACCTTGCCACCAGCT
CTCAAGTCACCAAACAAGCTCACCTCAACACCTCTGCTATCCTCTCCGTCGCCAGTTTGGCAAGAAAA
GCCCAAGTTGACTCTGACAACTCACACAACCAATACCCAGTTCATGCTTTCGGACCCCTGTCATCCAA
AAACAGCAAAGACATCACTGAAAGATACATCCCATACTTGGCCAACAAACTCAAGGAAGCTACCAGA
AACCAAGACAGTCTGAAAGCTCAAGTCTACATCAAAGCTCTCGGAAACTTAGGACATACCGCCGTCCT
CGCTGTCTTCAAACCATACCTTGAAGGAAAAGCCCCAGCCACCAACTTCCAACGTCTTTCAATGGTTG
CC

VgR sequencing base sequence, the sequencing result was basically consistent with the base sequence of *VgR* gene.

GATGGCAATGATGACTGTGGTGATAGATCCGATGAACAAGATTGTCCTAGCATAGATCTTACTGGGCAG
TGTCTTCCACCCAACTTCCTCTGTTCTACACTACCCAGCCTATGTCTACCTGCCAATGCCAAGTATGAGA
TGTCATGTGGTTCATGTGATACTCACTCTGAGTTTGAATGTCCTGAGTCACACCAATGCATACCCAACTC
CTGGCTATGTGACCATCAACCTGATTGTACTGGGGGAGAAGATGAGAACCCAGCACTCTGTACTCAGA
GGTACACTGCACCACGTCTATCATCTACTACACCCAGGTTTAGAGATGTGTTGCCATGTTCCGAGTTTT

The original template of *VgR* gene for synthesizing *dsVgR* (*Vitellogenin receptor*, *Diaphorina citri VgR*, 346bp).
TGTTCGATGACGATTGTCCTAGCATAGATCTTACTGGGCAGTGTCTTCCACCCAACTTCCTCTGTTCTAC
ACTACCCAGCCTATGTCTACCTGCCAATGCCAAGTATGAGATGTCATGTGGTTCATGTGATACTCACTCT
GAGTTTGAATGTCCTGAGTCACACCAATGCATACCCAACTCCTGGCTATGTGACCATCAACCTGATTGT
ACTGGGGGAGAAGATGAGAACCCAGCACTCTGTACTCAGAGGTACACTGCACCACGTCTATCATCTAC
TACACCCAGGTTTAGAGATGTGTTGCCATGTTCCGAGTTTTCCCAA

3 Supplementary Material Figures

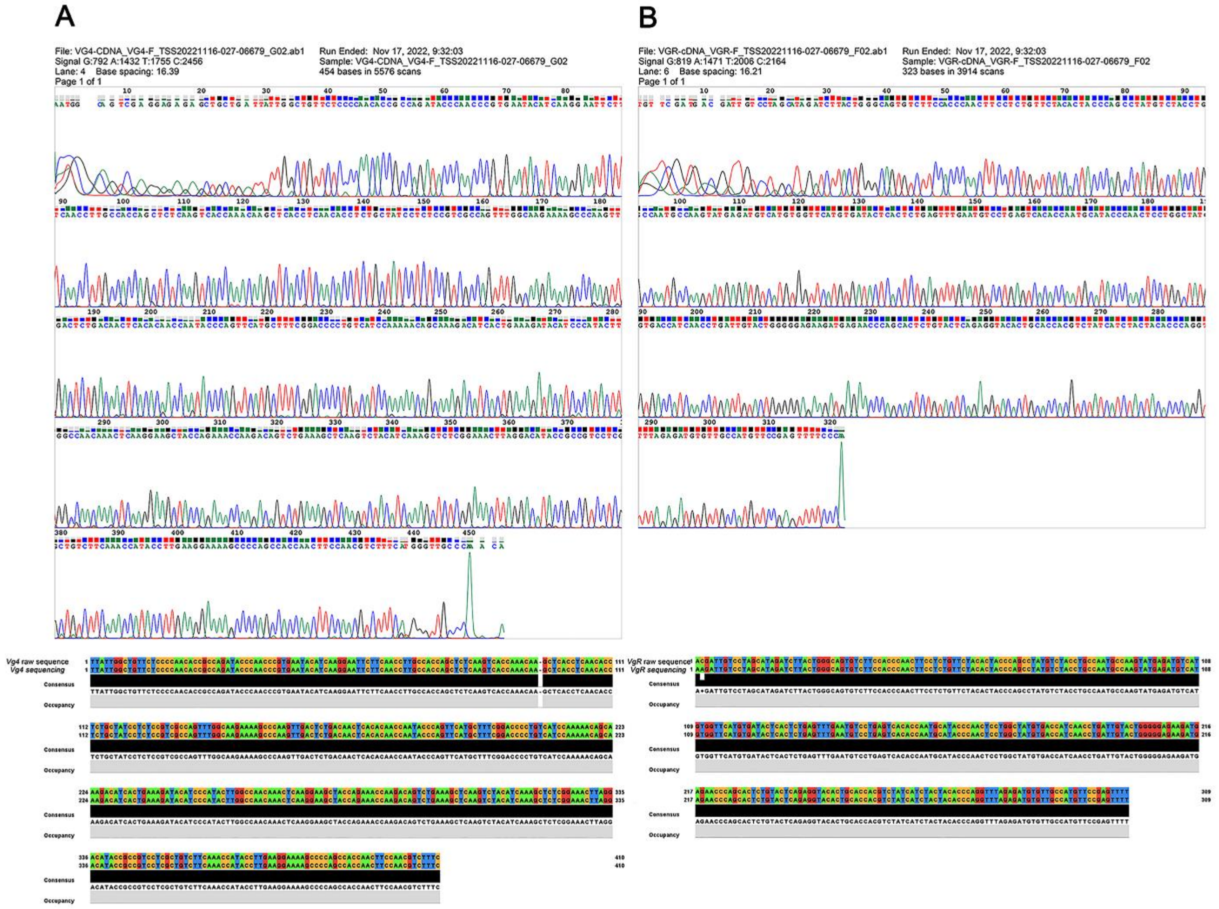

Figure S1. After *M. odorifera* shoots absorbed ds*Vg4* and ds*VgR*, total RNA was extracted and the sequencing results of *Vg4* and *VgR* gene interference fragments were cloned using cDNA as a template. (A) Base sequence and base feasibility of *Vg4* gene, sequence alignment between the *Vg4* sequencing sequence and the original sequence. (B) Base sequence and base feasibility of *VgR* gene, sequence alignment between the *VgR* sequencing sequence and the original sequence.

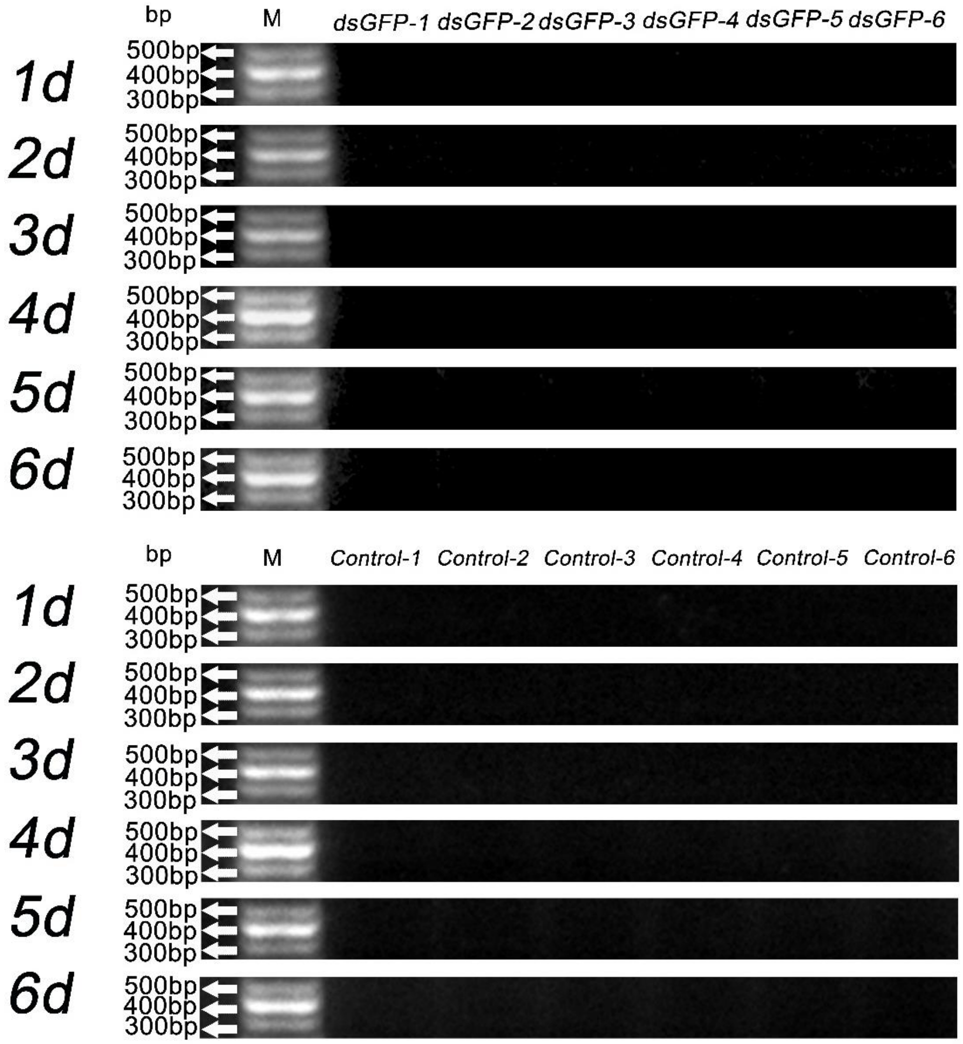

Figure S2. The ds*GFP* group and blank control group were detected by gel electrophoresis using *Vg4* and *VgR* PCR primers. The gel electrophoresis bands of ds*GFP* and blank control had no clear bands at 1-6 d. Ds*GFP*-1, 2, 3, 4, 5, 6/Control-1, 2, 3, 4 represent the six replicates for each treatment.

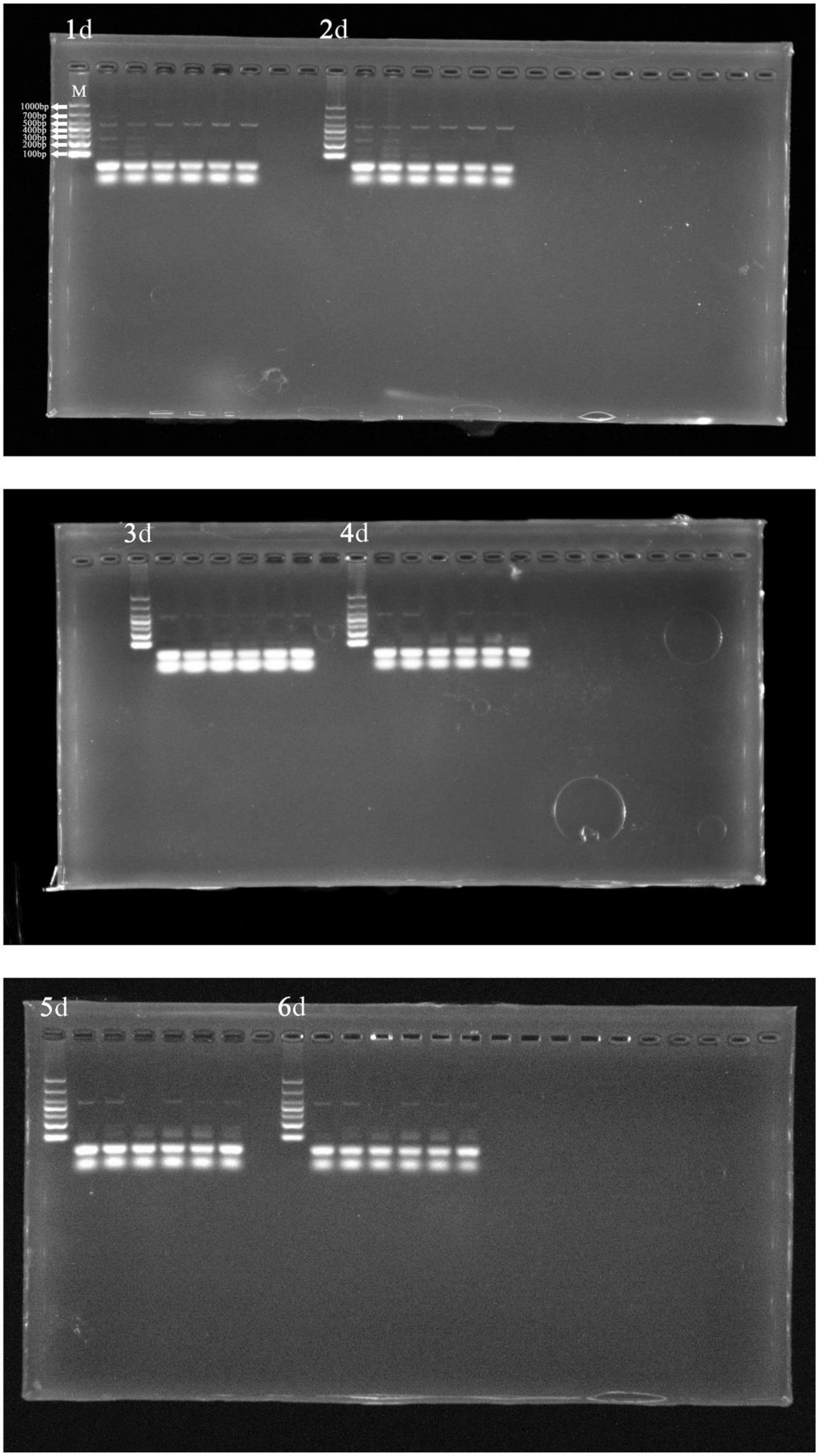

Figure S3. After ds*Vg4* was absorbed by tender *M. odorifera* shoots, the original gel electrophoresis was used to detect the persistence of ds*Vg4*. The original gel electrophoresis bands of ds*Vg4* were relatively clear at 1-6 d. The *Vg4* interference group treatment had six replicates.

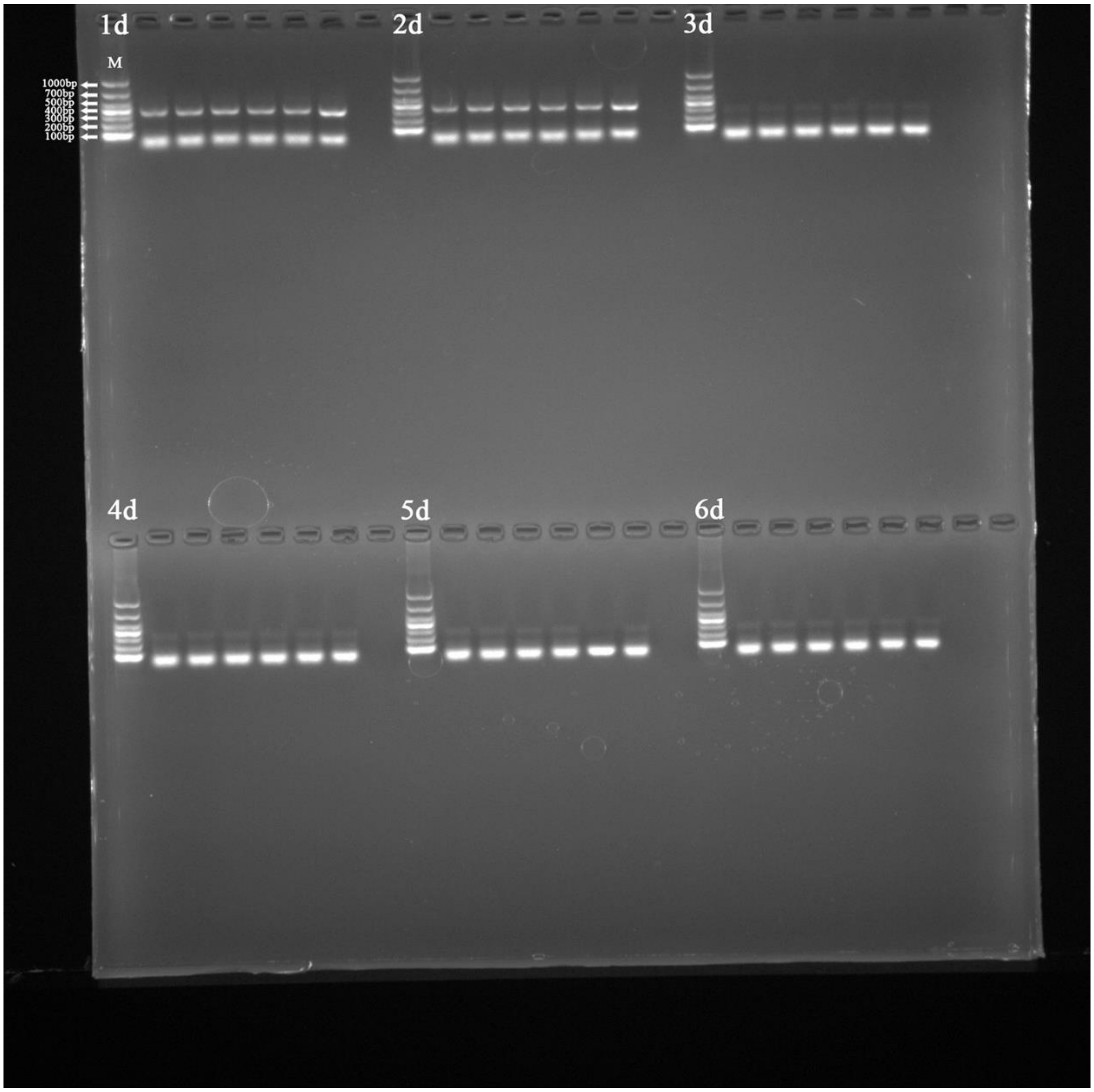

Figure S4. After ds*VgR* was absorbed by tender *M. odorifera* shoots, the original gel electrophoresis was used to detect the persistence of ds*VgR*. The original gel electrophoresis bands of ds*VgR* were relatively clear at 1-2 d, but blurry at 3-6 d. The *VgR* interference group treatment had six replicates.

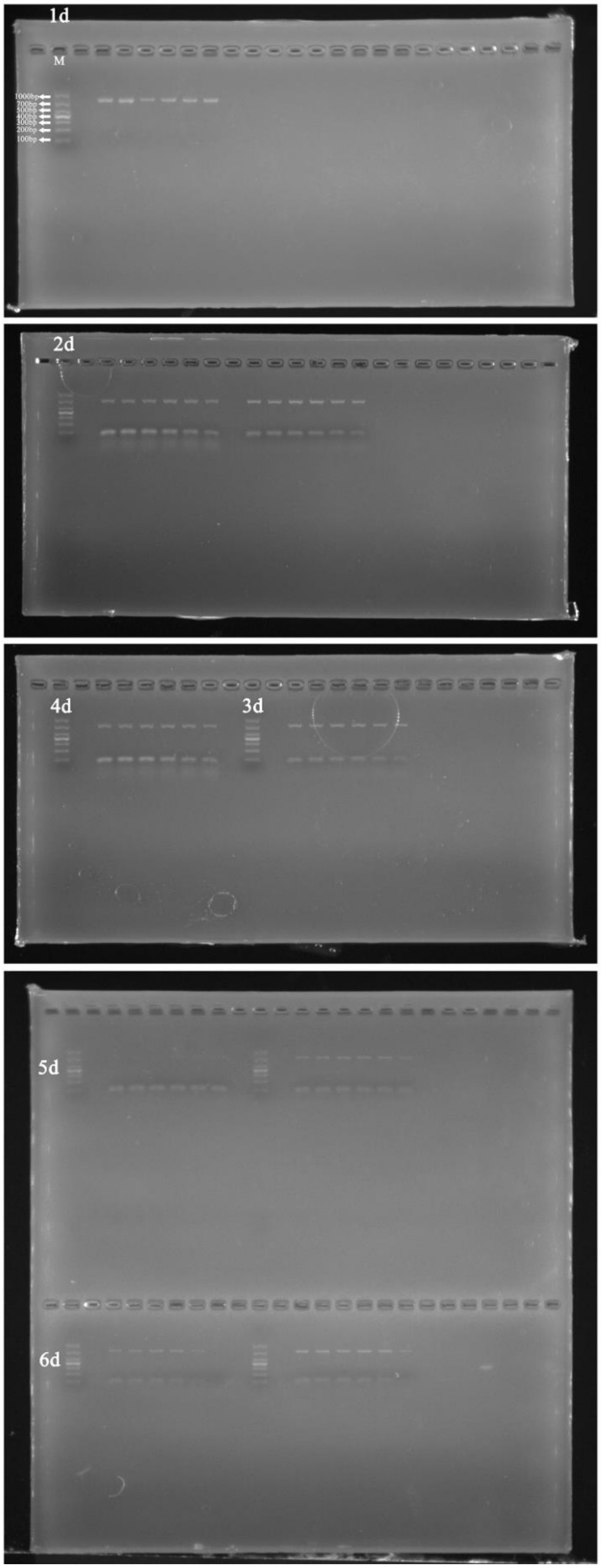

Figure S5. After ds*GFP* was absorbed by tender *M. odorifera* shoots, the original gel electrophoresis was used to detect the persistence of ds*GFP*, using *GFP* cloning primers for PCR. The original gel electrophoresis bands of ds*GFP* were relatively clear at 1-6 d. The ds*GFP* negative control group treatment had six replicates.

4 Full-length sequences of *Vitellogenin* genes and *Vitellogenin receptor* gene

LOC103523873 *Vitellogenin-1-like-1* [*Diaphorina citri* (Asian citrus psyllid)] Gene ID: 103523873, updated on 15-Jul-2019 1858 bp *Vg1*

>RNA-XM_008488883.3, gene id: LOC103523873, gene sequence

ATGGCTGTTGCCGCCACTGCTCAAGTTCCCCAACTTTCCGAAATGAACTTCATGAGATCTGAAAAGGA
AGACCCTGCCATGCAAATCGCCGCTGAAGCCATGTGGGGAGAGAATGCTCAATCTGGAGCTAAAATC
AGCATCAAGGCTAAACTCGAACAATCCGAACAACGCAAACAATACATTGCCAACCACCCACAAGCT
GAACAATGCAGAAAACAAATGGAACAAAGAGACTATGCTCTCAATGCTTGCCGCAACGTCACTGCTA
GATCCAATGCTCTTGATGAATACTCTCTCACCATCAAATACGAAAAGATCCCACAAAAGCTGATGAA
CGCAACCTACCAACTCTACAGAATCGCCCGTTACGCTGGATTTGCTTACAATTCTGAAAATGTTGTTG
CTGTTTCCAACCAAGCCGACCAATTGAAAGTCAGAGTGAACATTGCTGAAGACCACAAATCCGTCAA
TGTTTCCATTGAAGCCCCACACGCCAACTCTCAATTCAACAACCTCCCACTTTCCAACATGGCTAAAC
ACATCCTCATCCAAAACGCTCAATACGATGTAGACCAAAGAGTAGGATACGCTGCATTCAATGGCCA
ATACAACCCTGTGTGTGTTGCTGATGGATCATCCGGTCAAACCTTCGACAACAAGACCTACCCACTGA
ACCTTGAAAAAGACTCTTGGTACGTGCTCATGACTTCTGCTCCCAAACAAAGAAAAGACAACAGAGT
TGACTACAAAACCCAAAGACAAGAAAACGTCACCATCCTGGTCAAACAATCCGGAGAAAACAAAAA
GGAACTCAAAATCGTCTTGAACAACGGAGAACATGTTATTGACATGCAACCCTCTTCCTCCAACAAC
GATGGTGCCAACGCCAAAATCCAAGTCAACAAGAAAGACCAAAGAGCCTCCAAGAACAGCGTTACT
GAAGTCACCGACAACCAAAACCAAAGAATTGCTCAGATCTACGCTCTGCCCAGTGGAGAAGTCATCG
TCAACATGCCCAACCATGGACTTAGACTCAACTACGATGGAAGCCGCGTTCAAGTTCAAGCCACCGA
CCGTTTCAGAGACGGAGTTCGTGGACTCTGTGGATCTTTCACTGGAGAAAAAGCCACCGACTTCATCA
CCCCAAGAAACTGCATCGTCAGAGAAGCCAAAGACTTCGTTGCCACCTACTCCTTGTCCTCTAACAAC
AGAGACAACTCCAGACTCTCTAACCAAGACTTCTGTGCCCCCAAGAGACAAGTTCAATTCCAACAAG
TCATCAACGAAAAGAACATTGGACGTTGGGCTGACGCCAAACGATTGAATCTGCAAGGACTCTGGGG
ATTTTTCAACAAGAATGATGATGATGATAACAGCAGCAACAGCCAGAACAACTCTGGAAACAAGAA
CAACAGACGTAGCCCCAGACATGGAAACAACAGCCAAAACAGTAACGAAAACGACAGCAACAACC
AAAACAGCAATGAAAACAACAGAAACAACCAAGGCAGCGACGAGAACAACAAGAACAACCAACA
ACGTGGAAGTTCCAGCCACCGCCTCATGGTCGTTGAACAAGGAAACCAACTGTGCTTCAGTACCAAG
GCCATGCCCCAATGTAACCAAGGATACCGTGCTGAAAACACCGTTGAAAAGAAGGTACGTGATCACT
AA

>RNA-XM_008488883.3, gene id: LOC103523873, amino acid sequence

MAVAATAQVPQLSEMNFMRSEKEDPAMQIAAEAMWGENAQSGAKISIKAKLEQSEQRKQYIANHPQAEQ
CRKQMEQRDYALNACRNVTARSNALDEYSLTIKYEKIPQKLMNATYQLYRIARYAGFAYNSENVVAVSNQ
ADQLKVRVNIAEDHKSVNVSIEAPHANSQFNNLPLSNMAKHILIQNAQYDVDQRVGYAAFNGQYNPVCV
ADGSSGQTFDNKTYPLNLEKDSWYVLMTSAPKQRKDNRVDYKTQRQENVTILVKQSGENKKELKIVLNNG
EHVIDMQPSSSNNDGANAKIQVNKKDQRASKNSVTEVTDNQNQRIAQIYALPSGEVIVNMPNHGLRLNYD
GSRVQVQATDRFRDGVRGLCGSFTGEKATDFITPRNCIVREAKDFVATYSLSSNNRDNSRLSNQDFCAPKRQ
VQFQQVINEKNIGRWADAKRLNLQGLWGFFNKNDDDDNSSNSQNNSGNKNNRRSPRHGNNSQNSNEN
DSNNQNSNENNRNNQGSDENNKNNQQRGSSSHRLMVVEQGNQLCFSTKAMPQCNQGYRAENTVEKKV
RDH

LOC103523874 *Vitellogenin-1-like-2* [*Diaphorina citri* (Asian citrus psyllid)] Gene ID: 103523874, updated on 15-Jul-2019 1116 bp *Vg2*

>RNA-XM_008488884.1, gene id: LOC103523874, gene sequence

ATGCCCAACCATGGACTTAGACTCAACTACGATGGAAGCCGCGTTCAAGTTCAAGCCACCGACCGTT
TCAGAGACGGAGTTCGTGGACTCTGTGGATCTTTCACTGGAGAAAAAGCCACCGACTTCATCACCCCA
AGAAACTGCATCGTCAGAGAAGCCAAAGACTTCGTTGCCACCTACTCCTTGTCCTCTAACAACAGAG
ACAACTCCAGACTCTCTAACCAAGACTTCTGTGCCCCCAAGAGACAAGTTCAATTCCAACAAGTCATC
AACGAAAAGAACATTGGACGTTGGGCTGACGCCAAACGATTGAATCTGCAAGGACTCTGGGGATTTT
TCAACAAGAATGATGATGATGATGATAACAGCAGCAACAGCCAGAACAACTCTGGAAACAAGAACA
ACAGACGTAGCCCCAGACATGGAAACAACAGCCAAAACAGTAACGAAAACGACAGCAACAACCAA
AACAGCAATGAAAACAACAGAAACAACCAAGGCAGCGACGAGAACAACAAGAACAACCAACAAC
GTGGAAGTTCCAGCCACCGCCTCATGGTCGTTGAACAAGGAAACCAACTGTGCTTCAGTACCAAGGC
CATGCCCCAATGTAACCAAGGATACCGTGCTGAAAACACCGTTGAAAAGAAGATCGATGCCCACTGT
GTTCAAGACGGACAGTTAGCCAGACAATGGAAAGAACAAGCTCGCAGAGGTGAACACATCGCTGCC
ATGCAAAAGAAAAACCCCAACAAGACCATCACCGTTGAAGTACCAACCAAATGTGTTGCTGCCTAA

>RNA-XM_008488884.1, gene id: LOC103523874, amino acid sequence

MPNHGLRLNYDGSRVQVQATDRFRDGVRGLCGSFTGEKATDFITPRNCIVREAKDFVATYSLSSNNRDNSR
LSNQDFCAPKRQVQFQQVINEKNIGRWADAKRLNLQGLWGFFNKNDDDDDNSSNSQNNSGNKNNRRSP
RHGNNSQNSNENDSNNQNSNENNRNNQGSDENNKNNQQRGSSSHRLMVVEQGNQLCFSTKAMPQCN
QGYRAENTVEKKIDAHCVQDGQLARQWKEQARRGEHIAAMQKKNPNKTITVEVPTKCVAA

LOC103513507 *Vitellogenin-2-like* [*Diaphorina citri* (Asian citrus psyllid)] Gene ID: 103513507, updated on 15-Jul-2019 1197 bp *Vg3*

>RNA-XM_026826679.1, gene id: LOC103513507, gene sequence

ATGAACAAATATAATAAACTACTCAGTGAAAATCCAAGACTCCTTTACATCAAGGGGGTTCAAATCC
CGACCAAGAATGATGGAATTGTATCCTACAATATGACAACAGTTAAGAATCTGCAAGTCAGCCAAAA
CCTGCCAACCTGGGAACTGAACATCATCAAGAGTATTGTCAGTCAACTCCAAGTTGACACCAGAGCC
GAAAATGAAGTCAGCTCGCGTCTCAACCAGAAACCCAAGAACGGCAAACCTTTCGGAACCTTCAAG
ACCATGGAAGACACCGTCACTGGAGAATGTGAAACCTTGTACGACATCAAACCTCTGCAACAAAATC
GAATACCACTTCGGACTTCCCAAGTTGCCTTCAAGGTCGAACCTTTGAACAAGAACATGGAAGAGAA
AATCTTCATCGCCACCCAACACCCATACACCACCATCCAAAACATCCTCAACTTGACCCCAGCTGAA
AAATCACAAAACACCAAGGACATCAGCGTCAGAAAAACTAGAGAGTGGACCCAAACTTACGGAGA
ATCCAGCACTGGAGTTGCCATGAAAGTTCACTACAACGGTGAAGACAAAGCTGCTGACCTCGCATCC
TTCTTGAGATCAATGCAAGGTCATGACCTTACCTCCTACGCCTTGTTGAACAACTTCAACGTTGAAATC
AACAGACACAGATTCATTGTTTCCCACGATGCCCAAAAATCATCTGCCAAGGCTATTAGATTCTACGC
TAGCTACAGAAACAACGAAGAAAGCCGACTGAGAAACGCTGACAAAAACAACAATGACAATGCTA
ACAACAGTCCTCTTGAAGCTAGAGCTTCATCCGCTATTCCATCCACCCCATCCAAAAATGGAGCTGAA
AAACGCCAAGAAGAACTCTTACACAAAGCTACTTCTGGAATCAAAGATGCTGCCGCTACTGTAGTTG
AAATCCTGACCGAATTCGAAGGAAACAACAACGCTCAATATGTTTTGACTGCTGCCATTGCCAAGAG
CCCAGTTGACAAGAATTCCAAATTCCTGTTCTTCTACCATGCTTCTCCAGCTCAATCATCCAAATTTGA
AATGGCTGTTGCCGCCACTGCTCAAGTTCCCCAACTTTCCGAAATGAACTTCATGAGATCTGAAAAGG
AACTATATAACATCCACTGTCATTTCTGTCATTTAAAGAGGTGTGGGGA

>RNA-XM_026826679.1, gene id: LOC103513507, amino acid sequence

MNKYNKLLSENPRLLYIKGVQIPTKNDGIVSYNMTTVKNLQVSQNLPTWELNIIKSIVSQLQVDTRAENEVSS
RLNQKPKNGKPFGTFKTMEDTVTGECETLYDIKPLQQNRIPLRTSQVAFKVEPLNKNMEEKIFIATQHPYTTI
QNILNLTPAEKSQNTKDISVRKTREWTQTYGESSTGVAMKVHYNGEDKAADLASFLRSMQGHDLTSYALL
NNFNVEINRHRFIVSHDAQKSSAKAIRFYASYRNNEESRLRNADKNNNDNANNSPLEARASSAIPSTPSKNG
AEKRQEELLHKATSGIKDAAATVVEILTEFEGNNNAQYVLTAAIAKSPVDKNSKFLFFYHASPAQSSKFEMA
VAATAQVPQLSEMNFMRSEKELYNIHCHFCHLKRCGDI

LOC103523199 *Vitellogenin-3-like* [*Diaphorina citri* (Asian citrus psyllid)] Gene ID: 103523199, updated on 15-Jul-2019 4808 bp *Vg4*

>RNA-XM_026832896.1, gene id: LOC103523199, gene sequence

ATGAGCTCCGAACAAGGAAAGAACAGAAGAATCAGATCCCGTCGTTCTGTCATCAGAAACTCAAAC
GAAGACAACAACGAAAACAAGAACAACAAGAGACACAGTGACAATAACAGAGACAATGACTACAA
TGATGATGACCAAAAGAATCACCAAAACTCTGGATCCCACAACAACAACAACAACAACAACAACAA
CAACAACAACAATGACAACGGAAACAACAACTACAACAACAACGCCCGTGCATGGAAACAAGGAC
AAGTCTATGAATACCAAATCCAAGGCCGCACCCTAGCTGCTCTTCATGATGTAGCTGACCAATACACC
GGAACCATCATCAAAGCCACCCTCAAGGTTCAACCCAGAAATCAAGACTCAGTCTTGGCTTGGGTCA
CCAACGCTAGACACTCTGATGTTCACGCCAACCTAACCAATGGCTGGAACCAAGAGATCCCAGACAA
ATACCTCAACTACCAAAACTGGCAACTCAGTGACAAACCATTCGCCATCCAATTCAAGAATGGAGTT
GTTAAGAATCTGCAAGTCAGCCAAAACCTGCCAACCTGGGAACTGAACATCATCAAGAGTATTGTCA
GTCAACTCCAAGTTGACACCAGAGCCGAAAATGAAGTCAGCTCGCGTCTCAACCAGAAACCCAAGA
ACGGCAAACCTTTCGGAACCTTCAAGACCATGGAAGACACCGTCACTGGAGAATGTGAAACCTTGTA
CGACATCAAACCTCTGCAACAAGTTGAACAACAAAACAAACCTCAACTCGCCCCCATGCCAAACCTC
AAGGGATCAAACGGAGACCTTATTGACATCATCAAGACCAAGAACTTCAGTCGTTGTGACTCCAGAA
TCGAATACCACTTCGGACTTCCCGGAAGCAACGATGTTGAACCATCAAGCAACCAAGTTCTTAACTTC
TTGTCCAGATCTTCAACCAGCAGAGTCATCATTGCTGGAGACCTGAGCCACTACACCATCCAATCCTC
AGTCACCACCGACAAAATTGTCATCAGTCCCGAACTCTACAACAAACAAAAGGGAATGGTTGTCAGT
CGCCTGAACGTTACTCTGTCCAACGTCCACTCTGCTGCTCAAAACAATGCCCCAGCTCTCCCATCCAA
CGTCAACAAAGTTGATGATTTGATCTACGAATACAACCCAGCTTCCCCTGACAACGAATCTGCCCAA
AACAACAACGGCAAGAACAACCACCACAACGACGATGACTCATCCTCATCTTCTTCTTCAAGTTCTTC
CAGCTCCTCTTCTTCATCCTCCAGCAGTTCTGACAGTTCCTCCTCTTCTTCCTCGTCCTCCTCCTCAGAC
AGCTCATCATCTTCTGACAGTTCCAGCTCTTCCTCTGACAGCTCAAACTCTTCCTCCAACAGTTCCGAA
AGTGACGAGAGACAAAACAACAAGAGACGCAATAACAACAGCGAAGAAAACAACAAGAAGAACC
ACAACAACAGAAGCAACGAAAGTAGAAACAACAAAAACCACAATGGCAACAGCAATGAAAACAG
AAACAAGAACAACGAAGACAACGACTACACCATGAGCTCCGAACAAGGAAAGAACAGAAGAATCA
GATCCCGTCGTTCTGTCATCAGAAACTCAAACGAAGACAACAACGAAAACAAGAACAACAAGAGAC
ACAGTGACAATAACAGAGACAATGACTACAATGATGATGACCAAAAGAATCACCAAAACTCTGGAT
CCCACAACAACAACAACAACAACAACAACAATGACAACGGAAACAACAACTACAACAACAACGCC
CGTGCATGGAAACAAGGACAAGTCTATGAATACCAAATCCAAGGCCGCACCCTAGCTGCTCTTCATG
ATGTAGCTGACCAATACACCGGAACCATCATCAAAGCCACCCTCAAGGTTCAACCCAGAAATCAAGA
CTCAGTCTTGGCTTGGGTCACCAACGCTAGACACTCTGATGTTCACGCCAACCTAACCAATGGCTGGA
ACCAAGAGATCCCAGACAAATACCTCAACTACCAAAACTGGCAACTCAGTGACAAACCATTCGCCAT
CCAATTCAAGAATGGAGTTGTTAAGAATCTGCAAGTCAGCCAAAACCTGCCAACCTGGGAACTGAAC
ATCATCAAGAGTATTGTCAGTCAACTCCAAGTTGACACCAGAGCCGAAAATGAAGTCAGCTCGCGTC
TCAACCAGAAACCCAAGAACGGCAAACCTTTCGGAACCTTCAAGACCATGGAAGACACCGTCACTG
GAGAATGTGAAACCTTGTACGACATCAAACCTCTGCAACAAGTTGAACAACAAAACAAACCTCAACT

CGCCCCCATGCCAAACCTCAAGGGATCAAACGGAGACCTTATTGACATCATCAAGACCAAGAACTTC
AGTCGTTGTGACTCCAGAATCGAATACCACTTCGGACTTCCCGGAAGCAACGATGTTGAACCATCAA
GCAACCAAGTTCTTAACTTCTTGTCCAGATCTTCAACCAGCAGAGTCATCATTGCTGGAGACCTGAGC
CACTACACCATCCAATCCTCAGTCACCACCGACAAAATTGTCATCAGTCCCGAACTCTACAACAAAC
AAAAGGGAATGGTTGTCAGTCGCCTGAACGTTACTCTGTCCAACGTCCACTCTGCTGCTCAAAACAAT
GCCCCAGCTCTCCCATCCAACGTCAACAAAGTTGATGATTTGATCTACGAATACAACCCAGCTTCCCC
TGACAACGAATCTGCCCAAAACAACAACGGCAAGAACAACCACCACAACGACGATGACTCATCCTC
ATCTTCTTCTTCAAGTTCTTCCAGCTCCTCTTCTTCATCCTCCAGCAGTTCTGACAGTTCCTCCTCTTCTT
CCTCGTCCTCCTCCTCAGACAGCTCATCATCTTCTGACAGTTCCAGCTCTTCCTCTGACAGCTCAAACT
CTTCCTCCAACAGTTCCGAAAGTGACGAGAGACAAAACAACAAGAGACGCAATAACAACAGCGAAG
AAAACAACAAGAAGAACCACAACAACAGAAGCAACGAAAGTAGAAACAACAAAAACCACAATGG
CAACAGCAATGAAAACAGAAACAAGAACAACGAAGACAACGACTACACCATGAGCTCCGAACAAG
GAAAGAACAGAAGAATCAGATCCCGTCGTTCTGTCATCAGAAACTCAAACGAAGACAACAACGAAA
ACAAGAACAACAAGAGACACAGTGACAATAACAGAGACAATGACTACAATGATGATGACCAAAAG
AATCACCAAAACTCTGGATCCCACAACAACAACAACCATCACAACTCTGGATCTAATGACAACAAG
AACAACCATATGTCCAACAAAAACTGGAAACAAGGTCGCGATTCTTCCAGCTCCAGCTCTAGCTCTTC
TGACTCCAGCAGCTCTTCATCCAGCTCCTCAGAAAGTGGAAGTTCTTCTTCCTCTTCATCTTCTTCCTCC
AGCAGCAGCGAAGAATTCCACCAACCTGAACCCAAGCTTAACCAAGCCCCCAAATCTCCTTTCATGC
CCTACTTCATCGGAAACAAAGGAAACTCCATCGAATCTTCCAAACAAATCGACGGAGTTGCTGTCAC
CAAATCTTTGGCCCAACAAATTGGTCAAGAAATCCAAGAACCCAACACCTTGGCTGAACACAAAACC
CTTTCCAAATTCACCATCCTCGCCGGAGTTATCCGCACCATGAACGCCAAACAACTGGAAGCCGCCA
CTCACGAACTCTACTACCAACAAAACAAGGCCTCTTCATCCAGCCAATCCGATGCCACCAAATTAAC
CGCATGGAAAGCTTACCGTGACGCCGTTGCTCAAGCAGGAACTGGTCCCGCTTTGATGGCCATGAAA
CAATGGATTGAATCCGGCAAAGTCGAAGGAGAAGAAGCTGCTGAATTATTGGCTGTTCTCCCCAACA
CCGCCAGATACCCAACCCGTGAATACATCAAGGAATTCTTCAACCTTGCCACCAGCTCTCAAGTCACC
AAACAAGCTCACCTCAACACCTCTGCTATCCTCTCCGTCGCCAGTTTGGCAAGAAAAGCCCAAGTTGA
CTCTGACAACTCACACAACCAATACCCAGTTCATGCTTTCGGACCCCTGTCATCCAAAAACAGCAAA
GACATCACTGAAAGATACATCCCATACTTGGCCAACAAACTCAAGGAAGCTACCAGAAACCAAGAC
AGTCTGAAAGCTCAAGTCTACATCAAAGCTCTCGGAAACTTAGGACATACCGCCGTCCTCGCTGTCTT
CAAACCATACCTTGAAGGAAAAGCCCCAGCCACCAACTTCCAACGTCTTTCAATGGTTGCCGCCATG
GACCAAGTTGCCAGATTATCACCAAAATCTGTCCAACCTGCTCTCTTCAACATCTACCTCAACACCGG
AGAAAGCCACGAATTACGTTGTGCTGCTGTATTCCAACTCATGAAGACCTACCCATCCGCTCAATTGC
TCCAACGTATGGCTGCATTCACTGAACAAGACATGAGCAAACAAGTTAACTCTGCTGTCAAATCCGC
CATCGAAAGCGCTGCTGAACAACAACACCCCAAACTTCAAGAATTGTAA

>RNA-XM_026832896.1, gene id: LOC103523199, amino acid sequence

MSSEQGKNRRIRSRRSVIRNSNEDNNENKNNKRHSDNNRDNDYNDDDQKNHQNSGSHNNNNNNNNN
NNNNDNGNNNYNNNARAWKQGQVYEYQIQGRTLAALHDVADQYTGTIIKATLKVQPRNQDSVLAWVT
NARHSDVHANLTNGWNQEIPDKYLNYQNWQLSDKPFAIQFKNGVVKNLQVSQNLPTWELNIIKSIVSQLQ
VDTRAENEVSSRLNQKPKNGKPFGTFKTMEDTVTGECETLYDIKPLQQVEQQNKPQLAPMPNLKGSNGDLI
DIIKTKNFSRCDSRIEYHFGLPGSNDVEPSSNQVLNFLSRSSTSRVIIAGDLSHYTIQSSVTTDKIVISPELYNKQK
GMVVSRLNVTLSNVHSAAQNNAPALPSNVNKVDDLIYEYNPASPDNESAQNNNGKNNHHNDDDSSSSSS
SSSSSSSSSSSSSSDSSSSSSSSSSSDSSSSSDSSSSSSDSSNSSSNSSESDERQNNKRRNNNSEENNKKNHNNRSNES
RNNKNHNGNSNENRNKNNEDNDYTMSSEQGKNRRIRSRRSVIRNSNEDNNENKNNKRHSDNNRDNDY
NDDDQKNHQNSGSHNNNNNNNNNDNGNNNYNNNARAWKQGQVYEYQIQGRTLAALHDVADQYTG
TIIKATLKVQPRNQDSVLAWVTNARHSDVHANLTNGWNQEIPDKYLNYQNWQLSDKPFAIQFKNGVVKN
LQVSQNLPTWELNIIKSIVSQLQVDTRAENEVSSRLNQKPKNGKPFGTFKTMEDTVTGECETLYDIKPLQQVE
QQNKPQLAPMPNLKGSNGDLIDIIKTKNFSRCDSRIEYHFGLPGSNDVEPSSNQVLNFLSRSSTSRVIIAGDLS

HYTIQSSVTTDKIVISPELYNKQKGMVVSRLNVTLSNVHSAAQNNAPALPSNVNKVDDLIYEYNPASPDNES
AQNNNGKNNHHNDDDSSSSSSSSSSSSSSSSSSSSDSSSSSSSSSSSDSSSSSDSSSSSSDSSNSSSNSSESDERQNNK
RRNNNSEENNKKNHNNRSNESRNNKNHNGNSNENRNKNNEDNDYTMSSEQGKNRRIRSRRSVIRNSNE
DNNENKNNKRHSDNNRDNDYNDDDQKNHQNSGSHNNNNHHNSGSNDNKNNHMSNKNWKQGRDSS
SSSSSSSDSSSSSSSSSESGSSSSSSSSSSSSSEEFHQPEPKLNQAPKSPFMPYFIGNKGNSIESSKQIDGVAVTKSLA
QQIGQEIQEPNTLAEHKTLSKFTILAGVIRTMNAKQLEAATHELYYQQNKASSSSQSDATKLTAWKAYRDA
VAQAGTGPALMAMKQWIESGKVEGEEAAELLAVLPNTARYPTREYIKEFFNLATSSQVTKQAHLNTSAILS
VASLARKAQVDSDNSHNQYPVHAFGPLSSKNSKDITERYIPYLANKLKEATRNQDSLKAQVYIKALGNLGH
TAVLAVFKPYLEGKAPATNFQRLSMVAAMDQVARLSPKSVQPALFNIYLNTGESHELRCAAVFQLMKTYPS
AQLLQRMAAFTEQDMSKQVNSAVKSAIESAAEQQHPKLQEL

LOC113469177 *Vitellogenin-like* [*Diaphorina citri* (Asian citrus psyllid)] Gene ID: 113469177, updated on 10-Nov-2018, 1002 bp *Vg5*

>RNA-XM_026826680.1, gene id: LOC113469177, gene sequence

ATTTCAGCATGGAAACAAGGACAAGTCTATGAATACCAAATCCAAGGCCGCACCCTAGCTGCTCTTC
ATGATGTAGCTGACCAATACACCGGAACCATCATCAAAGCCACCCTCAAGGTTCAACCCAGAAATCA
AGACTCAGTCTTGGCTTGGGTCACCAACGCTAGACACTCTGATGTTCACGCCAACCTAACCAATGGCT
GGAACCAAGAGATCCCAGACAAATACCTCAACTACCAAAACTGGCAACTCAGTGACAAACCATTCG
CCATCCAATTCAAGAATGGAGTTGTTAAGAATCTGCAAGTCAGCCAAAACCTGCCAACCTGGGAACT
GAACATCATCAAGAGTATTGTCAGTCAACTCCAAGTTGACACCAGAGCCGAAAATGAAGTCAGCTCG
CGTCTCAACCAGAAACCCAAGAACGGCAAACCTTTCGGAACCTTCAAGACCATGGAAGACACCGTC
ACTGGAGAATGTGAAACCTTGTACGACATCAAACCTCTGCAACAAGTTGAACAACAAAACAAACCTC
AACTCGCCCCCATGCCAAACCTCAAGGGATCAAACGGAGACCTTATTGACATCATCAAGACCAAGAA
CTTCAGTCGTTGTGACTCCAGAATCGAATACCACTTCGGACTTCCCGGAAGCAACGATGTTGAACCAT
CAAGCAACCAAGTTCTTAACTTCTTGTCCAGATCTTCAACCAGCAGAGTCATCATTGCTGGAGACCTG
AGCCACTACACCATCCAATCCTCAGTCACCACCGACAAAATTGTCATCAGTCCCGAACTCTACAACA
AACAAAAGGGAATGGTTGTCAGTCGCCTGAACGTTACTCTGTCCAACGTCCACTCTGCTGCTCAAAAC
AATGCCCCAGCTCTCCCATCCAACGTCAACAAAGTTGATGATTTGATCTACGAATACAACCCAGCTTC
CCCTGACAACGAATCTGCCCAAAACAACAACGGCAAGAACAACCACCACAACGACGATGAC

>RNA-XM_026826680.1, gene id: LOC113469177, amino acid sequence

ISAWKQGQVYEYQIQGRTLAALHDVADQYTGTIIKATLKVQPRNQDSVLAWVTNARHSDVHANLTNGW
NQEIPDKYLNYQNWQLSDKPFAIQFKNGVVKNLQVSQNLPTWELNIIKSIVSQLQVDTRAENEVSSRLNQKP
KNGKPFGTFKTMEDTVTGECETLYDIKPLQQVEQQNKPQLAPMPNLKGSNGDLIDIIKTKNFSRCDSRIEYH
FGLPGSNDVEPSSNQVLNFLSRSSTSRVIIAGDLSHYTIQSSVTTDKIVISPELYNKQKGMVVSRLNVTLSNVHS
AAQNNAPALPSNVNKVDDLIYEYNPASPDNESAQNNNGKNNHHNDDD

LOC103524089 *Vitellogenin receptor* [Diaphorina citri (Asian citrus psyllid)] Gene ID: 103524089, updated on 15-Jul-20195950 bp *VgR*

>RNA-XM_026833263.1, gene id: LOC103524089, gene sequence

ATGGGTCCTTTGTATTTAGCAACGTGCTTCCTGTTTCTGCTTTACCCACCTTATCAACATGTGGTACGAG
GTGAAGATGTAACGTGTTTGGATAGTCAGTTCCTGTGTCACAACTCTACAGTGTGTATCGAGAAGTCT
CAAGTGTGTGATGGAGTTATACAGTGTCAATACGAAGACGATGAGTTTGATTGTGCTTTCTTCATGATT

GCAGATATCTACCTGTGTAAGTCGCCTAACTATTACAAATGTGAAGACGGCTCGTGTATAACATCCTC
CTTCTTGTGTGATGGAAGTGACGACTGTCTGGATGGTTCTGATGAAGCTAACTGTACGAGTAGTAGTG
CTCTCTCCAGTCAGCAAGAGTGTGATAGTATGGAATTCAAATGCAGCAATGGCAAGTGTATAACACA
ACATTGGGTGTGTGATGGGGAAGATGATTGTGGTGACGGGAGTGATGAAACTGAGATGGCGTGTAAG
AAGGTAGGTGACATCCTGTATATGAAGATTGATGGAGTTATACAGTGTCAATACGAAGACGATGAGT
TTGATTGTGATATCTACCTGTGTAAGTCGCCTAACTATTACAAGTGTGAAGACGGCTCGTGTATAACAT
CCTCCTTCTTGTGTGATGGAAGTGACGACTGTCTGGATGGTTCTGATGAAGCTAACTGTACGAGTAGT
AGTGCTCTCTCCAGTCAGCAAGAGTGTGATAGTATGGAATTCAAATGCAGCAATGGCAAGTGTATAA
CACAACATTGGGTGTGTGATGGGGAAGATGATTGTGGTGACGGGAGTGATGAAACTGAGATGGCGTG
TAAGAAGAACACGGATTGTATTGGGGACTTCCTATGTCGGAATCATAACTGTATAGTTAAGGATTGGG
TGTGTGATGGTGTCGATGATTGTCAGGATAATTCAGATGAAGAAAATTGTCCACACAAAGATGAGTTC
CCCATGTCGGAATGCACGTCAGAGCATCAGAGGTTCAAATGTCACGATGAAAGTCATTGTGTTGAGTT
TCATAAGTTATGTGATAATCATACTGACTGTATTGATGGATCTGATGAAGGGGGACAGTGCTCTACTG
TGTGTACGTATTCATGTAGGAAAAAATACTATCAACCTAGTTTCAAATGGAGCAACAAGTACAGCTTT
GATGGATTCAAGAGTTTTCCCGCGAGCTTTAAGATAGAAGATTGGAATGAGATGGAATTCATGCGGG
TGACAGCGGCTAAAGGTTACGCTCACATGGGACCTAACCCTTGTGCAGACTTTGGAGGGTGCAGTGA
TATTTGTCTATTGGCAGAAATAGTCGTGTCGGCAGGTCTAGGTTCCCCGGAAGACTTGTCTGTAGACTG
GCTCACACACAATATTTACTTCACGGATCTTAAGGCGCAACATATTGGAGTGTGCAATAATAAGGGA
GAGCATTGTGTGATCATAGTCAACGCAGATATAGATCGACCACGGGGTATAACGCTGCTCCCTGTAG
AACGCATAATGTTCTGGTCAGACTGGGGTAAAGTTCCCATGATAGCTACCAGCGGCATGGATGGCTCT
AACCCGCGACCTTTCATTAGCGACAGTATCCACTGGCCTAATGATGTCACTGTAGACTACTTCGGCTC
TAGACTGTACTGGATAGATGCTAAGCTGAAGATTATTGAAACTGTCAAGCTAGATGGAACTGACAGA
AGGACTGTTCTCTCGGATCGTGTCAAGCACCCATTTTCCATCGCCATCTTCGAAGACCAGATCTACTGG
AGTGATTGGGTTTCCATGGAGATATTGGCGTGTAACAAATTCACCGGTAGAGGGCGACATGTCATGCG
GAAAGAGAGAGATGATCAGATATATGGACTGCATGTTTACCATCCCGTCATGACTAATGTTCCTAATA
CTAGGAAAAGTACAATTTTAAAACTGACCATCATCATCAGTGATATCTCAGCTAAGAAGATCTCCTAT
CTGAATTTAGAGACCAGTGAGGTATCTCTCATACTACGAGGTTCAGTGGGTACTATCACTGCTATGGA
TTACGATAACCTGGGCTCTAACCTGTACTGGATTGATACGGAACGAGGTACTGTGGAGGTGTTGAATA
TGAAGAGACTTGTGCGTACCGTGCTACTACAGAACCTTACCGAGCTGCCTATCGCCCTTGCACTTGTG
CCTAAGGAAGGATTCATGTTCGTGGCATTCTCACACAAGCATCACATCCACATTGATCGTATTCGTAT
GGATGGCTCACTACAACAACGTACACATGTAATAGAGGATGGTTTAGTGGGACCTCACATTGTAATG
CACTATGATGAAGATTTAGAGCGAGTGTTCTGGGCAGATGCGTTCACTGGAGCTATAGAGAGTACTG
ATAGTCAAGGTCTGGACCGTCTCTCATACACCAATGTATCATCTCCCATGGGCTTGGCTACTGTTAGCA
ACGATCTATTCTGGACCAGCTATCAACCTAGTTTCAAATGGAGCAACAAGTACAGCTTTGATGGATTC
AAGAGTTTTCCCGCGAGCTTTAAGATAGAAGATTGGAATGAGATGGAATTCATGCGGGTGACAGCGG
CTAAAGGTTACGCTCACATGGGACCTAACCCTTGTGCAGACTTTGGAGGGTGCAGTGATATTTGTCTA
TTGGCAGGCAAAACACATGTATGCGCATGTCCTGCTGGTAAAATCCTCAATGCTAATGGTTTAACATG
TGAAGATCTGCCCAAGTGTTCCAATGAGAAACAGTTCCAATGTCATAATGGTCAATGTATAGCACTTC
ATCTGGTTTGCAACGGGCACAATGATTGTGTCGGTGGAGAAGACGAGCAAGCATCGTGCACGCCCTC
AGCACATCTGAATTGTTCTGAACTTCACTTCCCCTGTATGAACGGGGAGCGATGTATTGATCTAACGCT
CAGATGTAACAATGAGTTTGATTGTGAGGATAAATCTGATGAGTTCCATTGTAATAACACGGTCAAGA
CGTGCTCTGGAGAGTTTGATTTCCAGTGTGATTCAGGCGAATGTATTGGACGTCATTTCCTATGTGATA
AATCTAACGACTGTATGGATGGCTCAGATGAGAACCCTAAGCATTGTGTGAACTGTTCTAGTAGTGAG
TTTAGATGCGCTACAGGATCTTGTATACCTGCTAGCTGGATTTGTGATGGAGCCCCGGATTGTACAGA
CAATTCAGATGAGATGTATTGTGATAAGAAAGAAGTTTGTGGAACCTCAGCATTTACCTGTAGCAATG
AGAATTGTGTTCCTCTCAAGCTGAAATGTGATGGCAATGATGACTGTGGTGATAGATCCGATGAACAA
GATTGTCCTAGCATAGATCTTACTGGGCAGTGTCTTCCACCCAACTTCCTCTGTTCTACACTACCCAGC
CTATGTCTACCTGCCAATGCCAAGTATGAGATGTCATGTGGTTCATGTGATACTCACTCTGAGTTTGAA
TGTCCTGAGTCACACCAATGCATACCCAACTCCTGGCTATGTGACCATCAACCTGATTGTACTGGGGG

AGAAGATGAGAACCCAGCACTCTGTACTCAGAGGTACACTGCACCACGTCTATCATCTACTACACCC
AGGTTTAGAGATGTGTTGCCATGTTCCGAGTTTTCCTGTGAGAATGGTCAGTGTCTAAGATATTCACAA
GTGTGTGATAAACACCCTGACTGTATGGATGGTACAGATGAAGGTGGACGTTGCAACACTGGATGCT
CTACCATAGATTGTGACGTGTTTTATGAATTCTCGGTATGCGCATGTCCTGCTGGTAAAATCCTCAATG
CTAATGGTTTAACATGTGAAGATCTGCCCAAGTGTTCCAATGAGAAACAGTTCCAATGTCATAATGGT
CAATGTATAGCACTTCATCTGGTTTGCAACGGGCACAATGATTGTGTCGGTGGAGAAGACGAGCAAG
CATCGTGCACGCCCTCAGCACATCTGAATTGTTCTGAACTTCACTTCCCCTGTATGAACGGGGAGCGA
TGTATTGATCTAACGCTCAGATGTAACAATGAGTTTGATTGTGAGGATAAATCTGATGAGTTCCATTGT
AATAACACGGTCAAGACGTGCTCTGGAGAGTTTGATTTCCAGTGTGATTCAGGCGAATGCATTGGACG
TCATTTCCTATGTGATAAATCTAACGACTGTATGGATGGACCTCCTATGGAAGTTCTATACACAATGA
ATGATCAGCTTCGCAAAGTATCTTCCTCTCATCTCAAGATCATGTTTGAATATCCTGGAGTTCAGGTTA
AAGGTCTTGACATTGATATTAGAAAAGGGCTGGTCTATTGGTCGTCTGCAGAGTCAGGCATGGTCACT
CAGTTCAACATGAAAACTCTATCACGTCGCCTCTACATTTCCGGCCTTAGTCGTCCTGAGCGGCTCGC
ATTAGATTGGATTCGTAACCTAGTGTATATTGTAGAGTCAGAGAGGAAGATCATGGCGTGTCACATGG
AACGACATGTGTGCGTTAGGGTGTACTCTTCACTGGATAATATTCATATCTCTGCCCTCTCTGTAGATC
CTATCAATGGGTATTTATTCTGGGCGGAAACCTCCTGGCTGATGTGGGACGCACCAGTAGGTGTCATC
AAGCGTAGTGACCTCTCCGGTAGTAATGTGACGACAATAGTAGAAGGTAGCGTATCCCATGTGACCT
CAATCGCTATTAATGACATCAAGCGACATCTGTACTGGACAGACTCGGCTAAGAAACTAATAGAACA
GACTCAAATGGATGGAGCTTATAGGAAGAGGATACTAGATACCAAGGTCCCCGCACTTCAGTTGCAC
CTGTTCGAGGATATCCTATATTACGTGACGCCCATCATACAGGGTGCGCCATTCATCAAGTGCCACCT
CTATGGGAATCTACAGGGGTCTTGTGAGCAGCTGGATATACATGTGGCCAACCCTGTCACTCATTTCA
CCATATCACAATTGTCTAGACAGAGAATGGGTCCTAACAAGTGTACCAACTTCTCCTGTAGTCATATG
TGTTTAGAAAGTTCTACAGGGCCCGTGTGTATATGTCCTGATGGTAGTAAAGTCACCGGTAATCAGTA
CTGTGGAGTTAATATAGAGGATCCGATGTTAAGGAACCCTGCCATATTCCAGAACCATACCCAGGTG
GAGGGAGAGGATATGTCATCAGGTTCTGGTGTGGGTGTGCTGTTTATTATTGGTCTCATTCTAGTGGTG
CTCTCCATATCTGCAGTCTATCATTTGTGTCTCAGGAAACGAGTGGCAAACCTAGTACCCAAGATTCA
CTTCAGGAATCCCGTATTCAATGGAGGATACTCAGAGAGTAACTTAAGGAATGTTCCTATGAGTCAGA
ACTTCTATTCTCCTACCTCTCCTACTATGGTCTTACCTAATATTTATGAAAATGTAGTAGAGCCAGTCC
CTCCTGTTCAGATCAAGTATGCGAATGAGATGAGTATCAAACATGGAGAAGAAGATTTCTGGAATAC
TAGGAGACATTCAGAGTCCTCTACTGGTACTGATTATGCTGAGATACAAGACAACCCCAAACTACCTC
TCCTCTAG

>RNA-XM_026833263.1, gene id: LOC103524089, amino acid sequence

MGPLYLATCFLFLLYPPYQHVVRGEDVTCLDSQFLCHNSTVCIEKSQVCDGVIQCQYEDDEFDCAFFMIADI
YLCKSPNYYKCEDGSCITSSFLCDGSDDCLDGSDEANCTSSSALSSQQECDSMEFKCSNGKCITQHWVCDGE
DDCGDGSDETEMACKKVGDILYMKIDGVIQCQYEDDEFDCDIYLCKSPNYYKCEDGSCITSSFLCDGSDDCL
DGSDEANCTSSSALSSQQECDSMEFKCSNGKCITQHWVCDGEDDCGDGSDETEMACKKNTDCIGDFLCRN
HNCIVKDWVCDGVDDCQDNSDEENCPHKDEFPMSECTSEHQRFKCHDESHCVEFHKLCDNHTDCIDGSD
EGGQCSTVCTYSCRKKYYQPSFKWSNKYSFDGFKSFPASFKIEDWNEMEFMRVTAAKGYAHMGPNPCADF
GGCSDICLLAEIVVSAGLGSPEDLSVDWLTHNIYFTDLKAQHIGVCNNKGEHCVIIVNADIDRPRGITLLPVE
RIMFWSDWGKVPMIATSGMDGSNPRPFISDSIHWPNDVTVDYFGSRLYWIDAKLKIIETVKLDGTDRRTVLS
DRVKHPFSIAIFEDQIYWSDWVSMEILACNKFTGRGRHVMRKERDDQIYGLHVYHPVMTNVPNTRKSTILK
LTIIISDISAKKISYLNLETSEVSLILRGSVGTITAMDYDNLGSNLYWIDTERGTVEVLNMKRLVRTVLLQNLTE
LPIALALVPKEGFMFVAFSHKHHIHIDRIRMDGSLQQRTHVIEDGLVGPHIVMHYDEDLERVFWADAFTGAI
ESTDSQGLDRLSYTNVSSPMGLATVSNDLFWTSYQPSFKWSNKYSFDGFKSFPASFKIEDWNEMEFMRVTAA
KGYAHMGPNPCADFGGCSDICLLAGKTHVCACPAGKILNANGLTCEDLPKCSNEKQFQCHNGQCIALHL
VCNGHNDCVGGEDEQASCTPSAHLNCSELHFPCMNGERCIDLTLRCNNEFDCEDKSDEFHCNNTVKTCSG
EFDFQCDSGECIGRHFLCDKSNDCMDGSDENPKHCVNCSSSEFRCATGSCIPASWICDGAPDCTDNSDEMY

CDKKEVCGTSAFTCSNENCVPLKLKCDGNDDCGDRSDEQDCPSIDLTGQCLPPNFLCSTLPSLCLPANAKYE
MSCGSCDTHSEFECPESHQCIPNSWLCDHQPDCTGGEDENPALCTQRYTAPRLSSTTPRFRDVLPCSEFSCEN
GQCLRYSQVCDKHPDCMDGTDEGGRCNTGCSTIDCDVFYEFSVCACPAGKILNANGLTCEDLPKCSNEKQ
FQCHNGQCIALHLVCNGHNDCVGGEDEQASCTPSAHLNCSELHFPCMNGERCIDLTLRCNNEFDCEDKSD
EFHCNNTVKTCSGEFDFQCDSGECIGRHFLCDKSNDCMDGPPMEVLYTMNDQLRKVSSSHLKIMFEYPGV
QVKGLDIDIRKGLVYWSSAESGMVTQFNMKTLSRRLYISGLSRPERLALDWIRNLVYIVESERKIMACHMER
HVCVRVYSSLDNIHISALSVDPINGYLFWAETSWLMWDAPVGVIKRSDLSGSNVTTIVEGSVSHVTSIAINDIK
RHLYWTDSAKKLIEQTQMDGAYRKRILDTKVPALQLHLFEDILYYVTPIIQGAPFIKCHLYGNLQGSCEQLDI
HVANPVTHFTISQLSRQRMGPNKCTNFSCSHMCLESSTGPVCICPDGSKVTGNQYCGVNIEDPMLRNPAIFQ
NHTQVEGEDMSSGSGVGVLFIIGLILVVLSISAVYHLCLRKRVANLVPKIHFRNPVFNGGYSESNLRNVPMSQ
NFYSPTSPTMVLPNIYENVVEPVPPVQIKYANEMSIKHGEEDFWNTRRHSESSTGTDYAEIQDNPKLPLL

5 Normal ovarian structure and oogenesis process

According to the anatomical results of normal *D. citri* female reproductive system, ovary connected with the lateral oviduct and extends to the common oviduct. The end of the common oviduct connected with spermatheca, and eggs are stained with sperm through the end of common oviduct for fertilization. Colleterial gland are attached to the end of abdomen through colleterial gland tubes. *D. citri* female ovaries are juxtaposed left and right, ovariole is telotrophic type, each ovary has 18-21 ovariole, and most female have 20-21 ovariole. Ovariole varied among different individuals, sometimes 20 on one side and 21 on the other. Newly emerged female ovariole is short and translucent, ovariole length about 71.52 ± 19.91 μm and width about 20.93 ± 4.74 μm, no obvious egg chamber. Entering the oviposition period, ovariole length about 150.50 ± 23.83 μm and width about 26.17 ± 6.47 μm, with obvious egg chambers (**Figure 2-3**, **Figure 5-6**). The newly emerged female calyx is yellow, calyx diameter about 52.44 ± 4.05 μm. Entering the oviposition period, the calyx is obviously large and spherical, calyx diameter about 98.80 ± 8.48 μm.

D. citri female internal reproductive organs characteristics have changed significantly pass 5-30 d development (**Figure 5-6**). After 5 d development, the ovariole become dense and white, some ovariole turn yellow indicating that ovaries were forming eggs. At 5 d development stage, most females spermatheca surface is yellow and shriveled indicating that females don't mate with the males within 1-5 d, females are still in the pre-mating stage. After 10 d development, female ovaries began to form eggs, but the number of eggs was less indicating that most females didn't enter the oviposition period. At 10 d stage, female spermatheca become full and contained male's turbid sperm indicating that female entered the mating stage. After 15 d development stage, female ovaries had formed a certain number of eggs, the ovaries were gradually full and the number of eggs expands. At 15 d stage, ovaries color begins to turn yellow and female entering oviposition period. Due to most females enter mating period, the spermatheca becomes larger and contains more sperm than the 1-10 d females. After 20-25 d development, the female had more eggs in the ovary, and most female ovaries have more than 10 eggs. At 20-25 d stage, most female enter the peak oviposition period, ovariole and eggs are most dense compared with other periods, and ovaries volume is generally larger. At 20-25 d stage, most female spermatheca contain sperm. After 30 d development, although female ovaries were still a considerable amount of eggs, the amount of eggs and density of ovariole are decreased to a certain extent compared with 20-25 d female, and began to enter the oviposition decline stage. At 30 d stage, female spermatheca sperm load is reached the maximum.
