# Supplementary figures and images for: IPS (In-Plant System) Delivery of Double-Stranded Vitellogenin and Vitellogenin receptor via Hydroponics for Pest Control in Diaphorina citri Kuwayama (Hemiptera: Psyllidae)
Source: Int J Mol Sci. 2023 May 30;24(11):9497. doi: 10.3390/ijms24119497 (PMC10253278; doi:10.3390/ijms24119497)

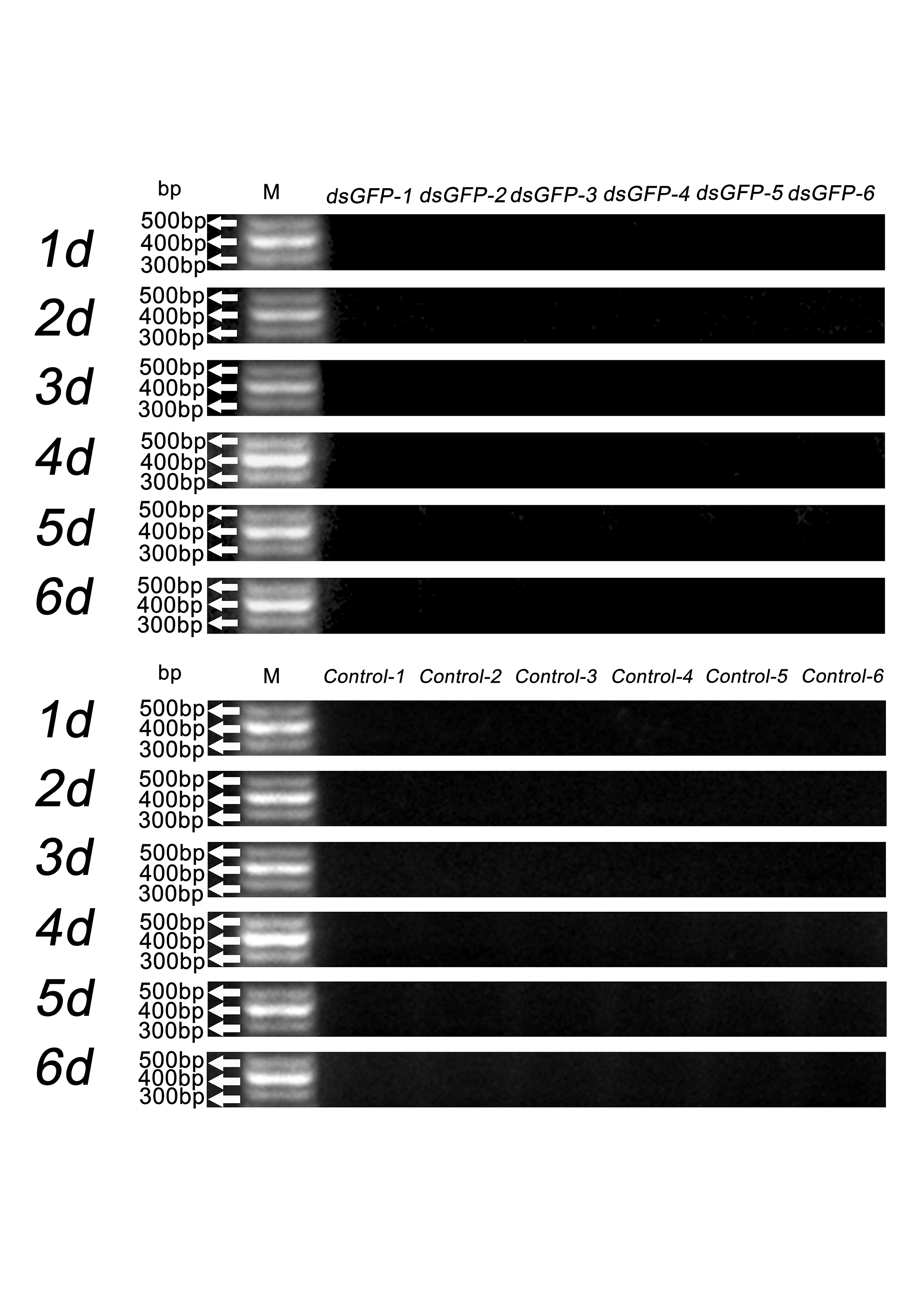

Supplement: Supplementary file 1 [file ijms-24-09497-s001.zip › Figure S2.tif]
